# The Book World of Medicine and Science

**Published:** 1898-03-12

**Authors:** 


### The Book World of Medicine and Science.

A Doctor of the Old School. By Ian Maclaren. With illustrations by Fred. C. Gordon. (London: Hodder and Stoughton. 1897. Price 2s. 6d.)

This is a book most suitable to the time in which we live—suitable by its very incongruity; for in the midst of the push and competition of professional life as we see it all around, in the midst of that struggle for place, for title, and for wealth which is so prominent a characteristic of latter-day medicine, is it not good to turn to a little history of a little glen buried in the highlands far away, to a story, the central figure of which is the doctor of the old school, who for a mere pittance, and at the expense of much hardship and many dangers—in fact, for no reward at all except the friendship and sympathy of those around him—" did his best for the need of every man, woman, and child in this wild, straggling district, year in, year out, in the snow and in the heat, in the dark and in the light, without rest and without holiday for forty years." The excellence, the pathos, the very teaching of a story lies in the telling thereof; and well as Ian Maclaren has done in many a larger work, he, probably, in none of them has succeeded better than in the little tale before us in rousing the interest and sympathy of his readers. Take the bald facts—the hardship, the poverty, the exposure, the constant, unceasing work—these may well make the young medical man, with his life before him, ask in disgust if this is all the reward his profession offers him; if this is all he has to hope for in return for his studies and his sacrifices in becoming qualified. But as Ian Maclaren tells the tale, the hard bitterness of the facts are lost in the wide human sympathy which pervades it, and for a moment one forgets the rattle of the life that is, and thinks, perhaps regretfully, of a life spent as was that of this doctor of the old school, and rewarded, as his was, by the love and honour and respect of all around.

Transactions of the British Institute of Preventive Medicine. First Series. (London: Macmillan and Co. 1898.)

This volume is a collection of papers written by members of the staff of the institute. In a short preface Lord Lister, the chairman of council, gives a brief historical account of the work done, and expresses his anticipation of still more fruitful labours in the future.

The first paper—by Dr. Bulloch—deals briefly with a point incidental to the controversy regarding the identity or non-identity of streptococcus erysipelatosus and S. pyogenes. Dr. Bulloch found that in one instance a horse immunised against a weak streptococcus was immune to another and virulent one. After the manner of bacteriologists, this experiment is made the basis of several confident generalisations.

Dr. Hewlett and Miss Knight add one more paper to the literature of the Klebs-Loeffler bacillus, and whittle away almost all distinctions yet left between the true and false bacilli of diphtheria. Dr. Symmons draws attention to a peculiar movement of intra-cellular particles that he has observed in yeast cells; and Mr. Foulerton, at some length, deals with the technique of the cultivation of the gonococcus, and recapitulates the pathology of gonorrhœa and its sequelæ in bacteriological terms.

Of the papers in this volume that have any real relation to preventive medicine the most valuable is that contributed by Drs. Macfadyen and Hewlett on the sterilisation of milk. Adopting the view, now so generally held, that Pasteurisation rather than sterilisation should be employed, they are led to recommend an ingenious method whereby all that Pasteurisation can accomplish is achieved in thirty seconds. The method, simply, is the circulation of the milk itself through coils of tubing immersed in hot water.

Mr. Lunt contributes a careful paper on the sterilisation of water by Berkefeld filters, and does not materially differ in his views from others who have paid attention to the question. The remaining papers deal with the bacteriology of plague, of dust in air, and the cultivation of water bacteria. This volume represents a large amount of careful work, but its perusal rather accentuates an existing feeling of disappointment with the results of bacteriology.

Cairo of To-day. By E. A. Reynolds, B.A., F.R.G.S. Maps and plans. 256 pages. (London: A. and C. Black. Price 2s. 6d. 1898.)

This is an excellent guide to a fascinating place, and is written with authority. It gives in a small compass a large amount of valuable information; it is nicely got up, well printed, and the maps are excellent. Many people now go to Cairo; some for health, some for novelty, some for society, and a few to see what ladies call "those dear pyramids." But every class of visitor will save much trouble and expense by taking to Cairo Mr. Reynolds' little book. The weakest part of it is that dealing with Egypt as a health resort. The chief element in the special therapeutic value of the Egyptian climate is the exceeding dryness of the atmosphere. And the practical outcome of this is that Egypt is one of the few places where patients in whom phthisis is complicated by albuminuria may hope to receive benefit. Other cases which are benefited by Egypt are those with bronchiestasis. These and other points would, perhaps, have been given greater prominence if the remarks on Egypt as a health resort had been revised by a medical man.

Fat and Blood: An Essay on the Treatment of Certain Forms of Neurasthenia or Hysteria. By S. Weir Mitchell, M.D. (Seventh edition. Philadelphia: T. B. Lippincott. 1898. Price 5s.; pp. 171.)

This latest edition of Dr. Weir Mitchell's classical essay has been carefully revised and, where necessary, added to. The treatment by means of rest, over-feeding, and massage, of certain forms of hysteria has, to use an Americanism, come to stay. Yet perhaps its value is hardly sufficiently recognised by the great body of practitioners. Weir-Mitchell treatment has, it is true, its disadvantages; it is costly, hurtful to the feelings of patient's friends, and the laity have a shrewd suspicion that it has in some quarters been unduly and improperly exploited. Yet with all this it remains by far the most effective weapon in the physician's hands for dealing with severe neurasthenia. But to be successful it must be carried out with scrupulous care; and, above all, the isolation from sympathising friends must be enforced. Dr. Weir Mitchell's wise, scholarly, and brilliant essay should be read by everyone who has not already become acquainted with it.

